# Ozone Detection via Deep-Ultraviolet Cavity-Enhanced Absorption Spectroscopy with a Laser Driven Light Source

**DOI:** 10.3390/s23114989

**Published:** 2023-05-23

**Authors:** Anthony Puga, Azer Yalin

**Affiliations:** Department of Mechanical Engineering, Colorado State University, Fort Collins, CO 80523, USA; aj.puga@colostate.edu

**Keywords:** ozone, ultraviolet spectroscopy, cavity-enhanced spectroscopy, deep ultraviolet

## Abstract

We present a novel sensing approach for ambient ozone detection based on deep-ultraviolet (DUV) cavity-enhanced absorption spectroscopy (CEAS) using a laser driven light source (LDLS). The LDLS has broadband spectral output which, with filtering, provides illumination between ~230–280 nm. The lamp light is coupled to an optical cavity formed from a pair of high-reflectivity (R~0.99) mirrors to yield an effective path length of ~58 m. The CEAS signal is detected with a UV spectrometer at the cavity output and spectra are fitted to yield the ozone concentration. We find a good sensor accuracy of <~2% error and sensor precision of ~0.3 ppb (for measurement times of ~5 s). The small-volume (<~0.1 L) optical cavity is amenable to a fast response with a sensor (10–90%) response time of ~0.5 s. Demonstrative sampling of outdoor air is also shown with favorable agreement against a reference analyzer. The DUV-CEAS sensor compares favorably against other ozone detection instruments and may be particularly useful for ground-level sampling including that from mobile platforms. The sensor development work presented here can also inform of the possibilities of DUV-CEAS with LDLSs for the detection of other ambient species including volatile organic compounds.

## 1. Introduction

Ozone (O_3_) is a critically important gas in the terrestrial atmosphere. Stratospheric ozone protects the Earth by absorbing most of the incoming ultraviolet (UV) radiation. Tropospheric ozone, unlike stratospheric ozone, is a harmful greenhouse gas and air pollutant and is designated by the 1970 Clean Air Act, from the United States Environmental Protection Agency (US EPA), as a criteria pollutant. Ozone is produced in the troposphere through sunlight-driven oxidation of volatile organic compounds, methane, and carbon monoxide [1]. High concentrations of surface-level ozone have adverse effects on human health, crop yield and quality, and natural vegetation [2,3]. Due to these concerns, and the status of ozone as a criteria pollutant, it is important to have reliable methods to measure and quantify emissions for regional air quality monitoring.

The US EPA classified the Denver Metro/North Front Range (DM/NFR) region in Colorado as a nonattainment zone for ozone in 1978 with the most recent rating of ‘Moderate’ in 2022 [4]. While ozone has multiple sources, including automobiles, increasing evidence links emissions from the oil and gas industry with ozone formation [5]. In particular, emissions of nitrogen oxide (NOx) species and volatile organic compounds (VOCs) from oil and gas activity are considered to be ozone precursors [6,7,8]. Within Colorado, there are currently in excess of 40,000 active oil and natural gas wells of which approximately one-third reside within Weld County in northern Colorado (part of the DM/NFR region). A recent study indicates that within Weld County, the oil and gas industry accounts for the highest ozone precursor emissions with 41% of the controllable NOx emissions and 78% of the controllable VOC emissions resulting from this sector [7].

The combination of US EPA regulatory requirements along with the concerns for human and vegetative health drives a strong need for reliable sensitive methods for ozone sensing. Our interests include mobile sensing, as has been developed for the detection of fugitive methane emissions, given that such approaches can provide a high rate of spatial coverage making them well-suited to field use, source apportionment studies, and the identification and quantification of mass balance emissions, e.g., in [9,10,11,12,13]. Sensors for such approaches should have high sensitivity, generally with detection limits to the order of ppb, which also allow a fast temporal response, generally with a response time of <~1 s. Note that throughout this paper, we use ppb and ppt to refer to volumetric (molar) fractions. Time response is of particular importance in mobile sampling since a slow instrument response can distort (delay and broaden) geolocated concentration readings. Two recent studies highlighted cases where instrument response time impacted the spatial profiles [14,15,16] recorded in mobile air quality studies. A response time of <1 s is generally considered reasonable for mobile sampling as this translates to relatively small distances, e.g., to <25 m for a vehicle speed of 50 miles per hour. It is also important to distinguish between the true instrument response time, i.e., the time required to respond to a step change in concentration, and the instrument readout time spacing (which can be arbitrarily configured). The availability of sensitive instruments with an even faster response time (>~10 Hz) can also enable direct flux measurements via eddy covariance (EC) analysis (see discussion in [17]).

To frame the interest in the sensor presented here, we first consider the landscape of available ozone sensors in the context of sensitivity and temporal response. Similarly to their use for detection of other trace atmospheric gases, semiconductor-based sensors tend to suffer from a combination of poor sensitivity, susceptibility to interferences and slow response time [18,19,20]. Commercially available semiconductor ozone sensors can have detection limits at the ppb (part per billion) level though generally come with response times limited to ~10–60 s. There has also been widespread development of optical instruments for ozone detection generally based on the principle of absorption spectroscopy, via the Beer–Lambert law, in the ultraviolet portion of the spectrum. Some of these instruments use lidar techniques to perform long-path-length measurements, often through atmospheric columns. For example, the travelling standard lidar operated by NASA’s Goddard Space Flight Center (NASA GSFC Stratospheric Ozone (STROZ) lidar), measures ozone based on the difference in returns of 308 nm light (strongly absorbed by O_3_) versus those of 355 nm light (much less absorbed by O_3_). The system uses high-power pulsed lasers (10 s of Watts) with a 76 cm receiver telescope to measure vertical profiles of O_3_ in the atmosphere over km length scales. Wing et al. discuss the use of the STROZ lidar and compare it with related instruments, including satellite-based approaches, for vertical column profiling [21]. In related work, Axellson et al. point to the use of different UV absorption wavelengths within the Huggins band (300–360 nm) versus that within the stronger Hartley band (200–300 nm), also considering absorption by other ambient species, again for relatively long path lengths (1.5 km), in this case with a fixed instrument based on differential optical absorption spectroscopy (DOAS) [22].

Our focus is on more compact optical instruments that make localized in situ (“point”) measurements and which can be used for mobile sampling deployments over smaller spatial scales. There are several instruments of this type, generally also based on absorption and typically targeting the strongest absorption region in the Hartley band. In many cases, the instruments use multi-pass cells or cavity-enhanced methods [23,24] to bolster sensitivity with light sources including mercury lamps (254 nm line), xenon lamps, and light emitting diodes (LEDs). For example, several commercial instruments based on UV absorption provide detection limits of ~3 ppb with response times of ~30 s. A research-grade instrument for O_3_ detection from a research aircraft, termed a fast photometer, using a mercury lamp (254 nm) was demonstrated by Gao et al. [25]. This instrument has a fast 2 Hz sampling rate, an accuracy of 3–5%, and a precision of ~3 ppb. A relatively similar instrument, again using the 254 nm output from a mercury lamp, and oriented toward sampling from stratospheric balloons has also been shown [26]. 

Another sensitive optical detection approach, shown by Washenfelder et al., detects O_3_ via the conversion of NO (which must be carried with the sensor) into NO_2_, subsequently allowing the detection of NO_2_ at 404 nm with cavity ring-down spectroscopy (CRDS) [27]. The approach allows O_3_ detection via a differencing method with a (2 σ) detection limit of ~0.13 ppb and is partly motivated by better CRDS mirror reflectivity and component availability in the visible region of the spectrum. Related work has leveraged the relatively recent availability of high-brightness DUV LEDs that can access the Hartley band [17,28,29]. Hannun et al. have developed the rapid ozone experiment (ROZE) which is a broadband cavity-enhanced absorption spectroscopy (CEAS) UV instrument for the point detection of O_3_ using an incoherent 265 nm LED light source allowing a precision of ~30 ppt in 1 s [17]. The performance specifications of the instrument are impressive though it is noted that accessing other molecules in nearby spectral regions, particularly at even shorter wavelengths where many related VOC species are absorbed, may be limited by LED availability.

The present contribution examines ozone detection via broadband CEAS but, for the first time, using a xenon laser-driven light source (LDLS) to provide the UV illumination. The xenon LDLS consists of a diode or fiber laser which delivers a focused beam to the center of a xenon bulb, thereby producing a small luminous plasma which emits over a very broad spectral region spanning ~190 nm–2.5 mm. These sources are relatively new and are being considered in a range of applications due to their several attractive features: very broad wavelength coverage including the deep-ultraviolet range (DUV), high spectral brightness also including high spatial coherence (fiber-coupled output is possible), and, from a practical perspective, a long lifetime of >~100,000 h [30,31,32]. From the perspective of the present instrument, the high spectral brightness in the ~230–280 nm region is critical, but so too is the high spatial coherence, which allows fiber output and, ultimately, the efficient coupling of the LDLS output light to an on-axis optical cavity. The use of the small-volume optical cavity is important with regard to being able to achieve relatively fast gas residence (flush) times with compact pumps, thereby enabling a fast temporal response. There have been limited reports on the use of LDLS for spectroscopic gas sensing with the work of Washenfelder et al. being a standout example of a CEAS instrument for the detection of formaldehyde at ~315–350 nm [33]. Our work follows some of the methodology developed in said study.

In this work, we demonstrate CEAS at DUV wavelengths with the LDLS for the sensitive measurement of ozone. The CEAS instrument described in this work is suitable for laboratory deployment as well as field deployment on a mobile cart and allows ppb level detection with fast response times (<1 s). The principle of operation as well as the setup of the CEAS instrument are presented in Section 2. The results of laboratory testing for accuracy and precision, along with initial field testing, are provided in Section 3. Finally, conclusions and avenues for future research are presented in Section 4.

## 2. Experimental Methods

### 2.1. Experimental Setup

**Figure 1:**
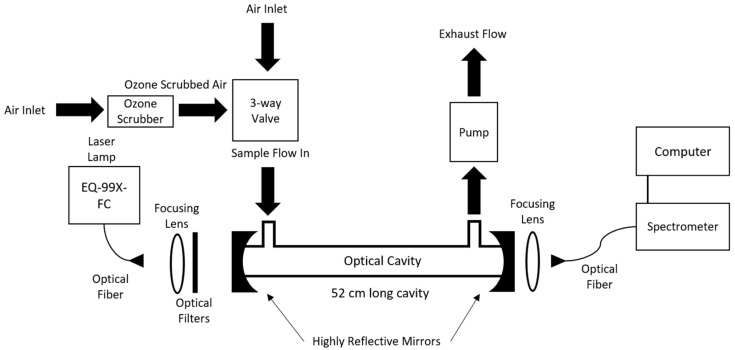
Experimental setup for ozone sensor with laser-driven light source (LDLS) and broadband cavity-enhanced cavity absorption spectroscopy.

The setup for the CEAS ozone sensor is shown in Figure 1. The overall instrument comprises a DUV LDLS, collimation optics, spectral filters, an optical cavity, and a spectrometer detector. The LDLS is a broadband laser-driven Xenon arc lamp (EQ-99X-FC LDLS Energetiq, Wilmington, MA, USA) comprising a fiber-delivered continuous-wave diode laser that drives a small and highly luminous Xe plasma [34]. The lamp is air-cooled and only requires current during the plasma ignition phase, thereby allowing extended operation. The Xe plasma generates optical emission (output light) across a broad spectral region of ~190–2500 nm. The light is collected within the lamp and delivered by a solarization-resistant UV-rated optical fiber of a 1 m length, 230 mm core diameter, and numerical aperture (NA) of 0.22, for the experiment. The light power exiting the fiber has some variation across our range of interest (~230–280 nm) but is approximately 30 mW/nm at these wavelengths, corresponding also to a spectral radiance of ~10 mW/mm^2^/sr/nm at the fiber output. The combination of high power, but also high spatial coherence (ability to fiber couple), gives a final radiance value that is significantly higher than that from many competing sources including xenon or deuterium lamps, thereby supporting the suitability of the LDLS. The light exiting the lamp output fiber is collimated with a focusing lens and then spectrally filtered to a smaller band of ~230–280 nm with a 3-filter stack containing a 225 nm long-pass filter (Newport, 10CGA-225, Irvine, CA, USA), 276 nm short-pass filter (AVR Optics, FF01, Fairport, NY, USA), and 265 nm short-pass filter (Asahi Spectral, XUS0265, Torrance, CA, USA). While in principle light at other wavelengths should not affect the UV absorption, filtering helps to reduce stray light effects at the final detection stage. The filtered light is then coupled to an on-axis optical cavity formed by two mirrors (diameter of 2.54 cm, radius of curvature of 1 m, and peak reflectivity at ~248 nm) with a peak reflectivity of ~0.99 in our region (see also Section 3.1). The mirrors are mounted on both ends of a 52 cm long stainless-steel cell with a 3.5 cm inner diameter and with gas ports for input and output (cell volume of ~0.50 L). To illustrate the possibility of a fast response time we have also carried out some measurements with a smaller cavity diameter of 1.05 cm and length of 40 cm (cell volume of ~0.034 L). 

Light exiting the cavity is coupled with a 5 cm focal length lens to a 600 mm core 2 m length UV/VIS fiber which relays the cavity output light to a grating spectrometer (Maya 2000 Pro OceanInsight, Orlando, FL, USA). The spectrometer is optimized for DUV operation with a back-thinned charge coupled device with wavelength range of 200–345 nm. The spectrometer has a fixed 25 mm input slit with 1800 groove/mm grating resulting in a spectral resolution of 0.17 nm. A custom LabVIEW program records the spectral output for given integration times. The spectrometer is typically run at a 1 s integration (reporting) time for the acquisition of spectra, with overall collection times for the sample and reference legs of either 5 min sample/1 min reference or 10 min sample/1 min reference.

Air is drawn through the cavity by a pump on the back end of the cell with a flow rate of 12.5 lpm (as measured by a rotameter). The pump is mounted in a custom heat sink to maintain quiet and steady performance for long tests. This flow rate corresponds to a Reynolds number of ~500 (and linear velocity of ~20 cm/s) within the flow cell and is not expected to introduce turbulence. The measurement procedure is based on sequentially switching between a sample gas (containing O_3_) and reference air gas (without O_3_) with pairs of these readings being used in a ratio method (see below). The flow switching is controlled by a custom LabVIEW program driving a 3-way solenoid switch. In some cases, we use a cylinder of ultra-pure zero air for the ozone-free reference, while in other cases we use a scrubber (carulite filter from 2B Tech, Broomfield, CO, USA) to remove ozone from the air stream. Data from the first 15 s after valve switching are discarded to accommodate for switching and flush times.

We have performed some experiments to study sensor accuracy with a commercial ozone calibration source (Model 306 Ozone Calibration Source 2BTechnologies). This unit allows a selectable ozone concentration in the range of 30–1000 ppb. The ozone calibration source is also capable of producing ozone-free air by shutting off the lamp used to produce the ozone molecules. The ozone calibration source used has a manufacturer-stated precision of (the higher of) 2 ppb or 2% of the ozone concentration setting. During measurements, we allowed the ozone calibration source to stabilize each time the output was changed based on the manufacturer-recommended time of 30 s.

### 2.2. Principle of Operation and Analysis Method

**Figure 2:**
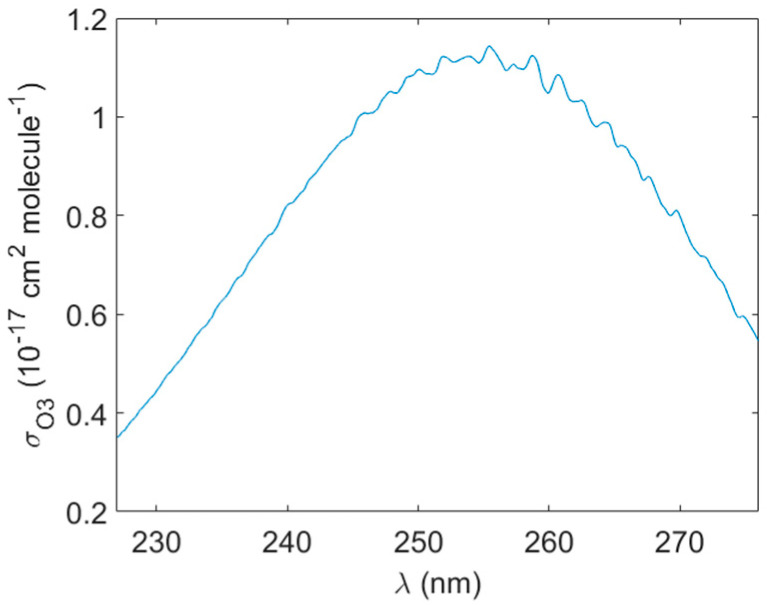
Ozone absorption cross-section in spectral region of interest from [35].

Ozone concentrations from air samples are measured in a ratio-based approach using the recorded spectra where we denote the sample and reference zero-air (ozone-free) signals as *I*_O3_(*l*) and *I_ZA_*(*l*), respectively. We follow the derivation of Washenfelder et al. [33] to find the ozone absorption coefficient (per unit length), *k*_O3_, which is defined as the product of the ozone cross-section, *s*_O3_ (units of area), with the ozone number density, *n*_O3_ (units of inverse volume):

(1) $$k_{O3}\equiv \sigma {\left(\lambda \right)}_{O_{3}}n_{O_{3}}=\left(\frac{I_{ZA}\left(\lambda \right)}{I_{O3}\left(\lambda \right)}\right)\left(\frac{1-R\left(\lambda \right)}{d}+\alpha {\left(\lambda \right)}_{ZA}\right)$$

where *R*(*λ*) is the wavelength-dependent mirror reflectivity, *d* is the physical cavity length, and *a*(*λ*)*_ZA_* is the wavelength-dependent extinction of reference air (primarily due to Rayleigh scattering; separately calculated). Determination of ozone number density (and then concentration) from the measured spectrometer signals requires knowledge of profiles of mirror reflectivity and the O_3_ absorption cross-section. For the latter, we use published results from Molina et al. [35]. Figure 2 shows a plot of the O_3_ absorption cross-section in the wavelength range of interest. Note that these data were obtained with a resolution of 0.5 nm which matches reasonably well with the 0.17 nm wavelength resolution of our system (and the mismatch is unimportant given the spectrally broad profile). 

Precise determination of the mirror reflectivity profile can be achieved by measuring CEAS signal differences due to the introduction of a gas with well-known Rayleigh scattering and/or absorption, again referenced against zero air [33]. In the present system, we generally employ a known O_3_ concentration (from the O_3_ generator) referenced against ozone-free zero air (at the same overall density and pressure). The reflectivity profile can be determined through the following relation:

(2) $$R\left(\lambda \right)=1-d\left(\frac{I_{ZA}\left(\sigma {\left(\lambda \right)}_{Ray}n_{ZA}\right)-I_{O_{3}}\left(\sigma {\left(\lambda \right)}_{Ray}n_{ZA}+\sigma {\left(\lambda \right)}_{O_{3}}n_{O_{3}}\right)}{I_{O_{3}}\left(\lambda \right)-I_{ZA}\left(\lambda \right)}\right)$$

where *σ*(*λ*)*_Ray_* is the air Rayleigh scattering cross-section [36], and *n_ZA_* is the number density of air. Known concentrations of ozone in air, along with the ozone-free zero air, were produced using the ozone calibration device discussed above (note that the calibration approach assumes the same overall air density for both ozone and zero air, as is the case here). Experimentally, we find that the reflectivity profile generally remains unchanged over periods of several weeks or more. 

The present analysis method accounts for O_3_ as the only chemical absorber given that the region is quite free of significant interferences from other ambient species. Of the main ambient species, the known interferants are water and oxygen. In the case of water, the strongest line intensity in our spectral region (~230–280 nm) is only ~5 × 10^−28^ cm/molecule [37], corresponding to absorption coefficients of several orders of magnitude below our detection limit for even 100% relative humidity. Oxygen has weak absorption in our spectral region due to the Herzberg band with a cross-section of ~2 × 10^−24^ cm^2^/molecule [38] at the peak of the O_3_ spectrum (*l*~255 nm). This value is weaker than that of O_3_ by a factor of ~5 × 10^6^ meaning that O_2_ absorption will remain small (relative to O_3_) for O_3_ concentrations as low as ~40 ppb (see also Section 3.2). 

## 3. Results and Discussion

### 3.1. Mirror Reflectivity and Cavity Spectral Throughput

**Figure 3:**
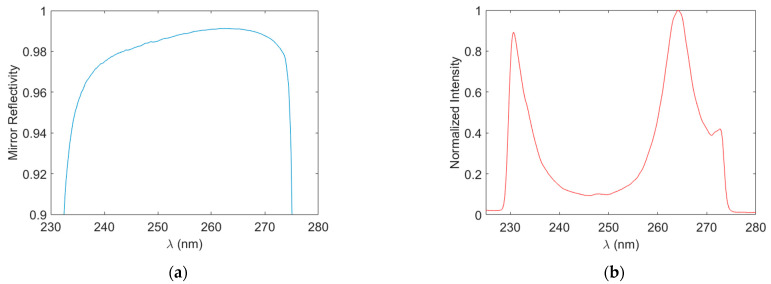
Spectral profiles of cavity mirror reflectivity (**a**) and cavity output light (**b**).

Figure 3a shows the wavelength-dependent mirror reflectivity calculated with Equation (2). We find a peak mirror reflectivity of 99.1% at 262 nm with reflectivity exceeding 98.8% over the range of ~243–273 nm. Mirror reflectivity in the DUV is inevitably poorer than that in the visible and infrared regions. The obtained results are reasonable and correspond to a maximum effective path length of ~58 m. However, past CRDS work in our group, with these mirrors, yielded R~99.7% suggesting that we may have room for improvement in this area [39]. Figure 3b shows an example of the light spectrum recorded after the cavity obtained at a 1 s integration time with the cavity filled by zero air. The spectral profile of intensity is due to the product of the output of the LDLS source (including fiber), the optical filter stack, and the cavity transmission profile. The final light spectrum has a low intensity across ~240–255 nm since this coincides with the peak reflectivity of the cavity mirrors (centered at ~248 nm). The strong intensity falloff below ~230 nm, and above ~275 nm, is due to pre-cavity filtering and serves to reduce light contamination within the spectrometer.

### 3.2. Sensor Accuracy for Ozone Detection

**Figure 4:**
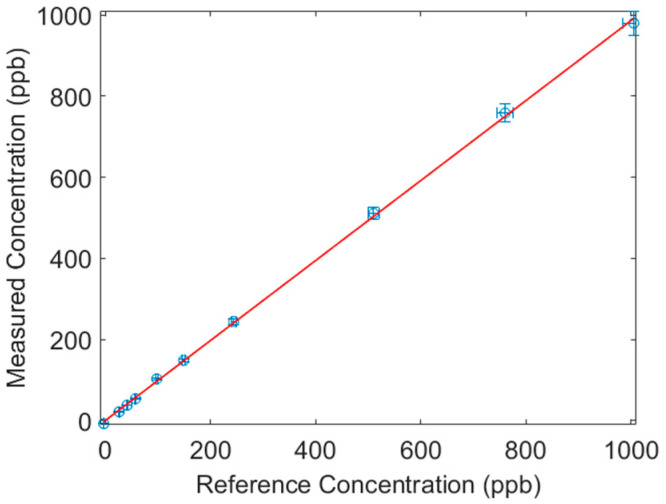
Study of measured O_3_ concentration versus reference concentration to examine sensor accuracy.

The accuracy of the DUV-CEAS sensor was examined by comparing its readings against those of a commercial federal equivalent method ozone monitor (Model 202 Ozone Monitor 2BTechnologies, Broomfield, CO, USA) using fixed concentrations from the ozone calibration source. The inlets of the DUV-CEAS instrument and reference monitor were connected to the outlet of the ozone calibration source for simultaneous measurements of the same stream in the range of 0–1000 ppb. For the data in Figure 4, both instruments recorded the ozone concentration for 5 min at each fixed value. The uncertainty value (horizontal error bar) for reference concentrations is the higher of 2 ppb or 2% of the setting, so 2 ppb for settings ≤100 ppb and 2% is for higher settings. Uncertainly in the DUV-CEAS is plotted as 3% based on the standard deviation of the readings. Clearly, we see a high accuracy better than ~2%, based on the good agreement of our DUV-CEAS instrument versus that of the commercial reference analyzer (slope of 0.986 and R^2^ value of 0.9997). Note that the instrument maintains high accuracy even at O_3_ levels of only 10 s of ppb where O_2_ absorption is non-negligible. This positive result is attributed to the spectrally resolved fitting method over a relatively broad (~30 nm) range making the instrument robust against spectrally structured interferences (i.e., O_2_) present over limited spectral regions.

### 3.3. Sensor Precision for Ozone Detection

**Figure 5:**
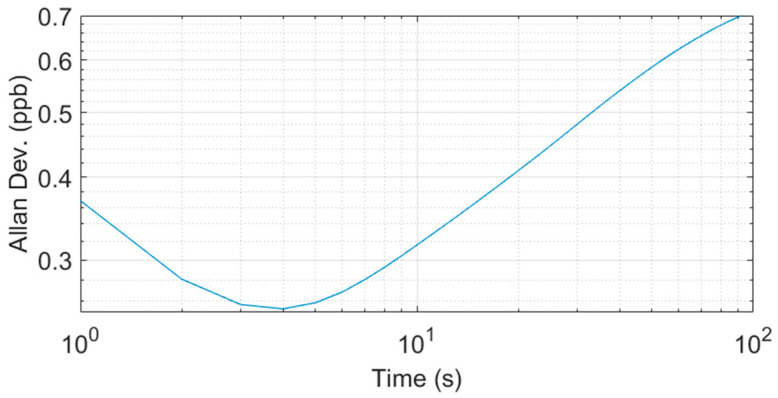
Allan deviation analysis of ozone concentration recorded with DUV-CEAS instrument.

The Allan variance method provides an accepted and consistent method to characterize the stability and precision (limit of detection) of optical instruments [40,41]. Following standard methods in the field, we determine the sensor precision based on the reading of zero concentration, in this case by filling the cavity with pure nitrogen (99.999%). Ozone concentration readings were taken at a rate of 1 Hz. Figure 5 shows the Allan deviation (ppb) plotted against the measured duration. The Allan deviation has a minimum of ~0.3 ppb (for 4 s integration time) while also yielding ~0.4 ppb for a 1 s integration time. The sub-ppb result indicates the utility of the sensor for practical field measurements related to source identification and emission detection.

### 3.4. Response Time for Ozone Detection

**Figure 6:**
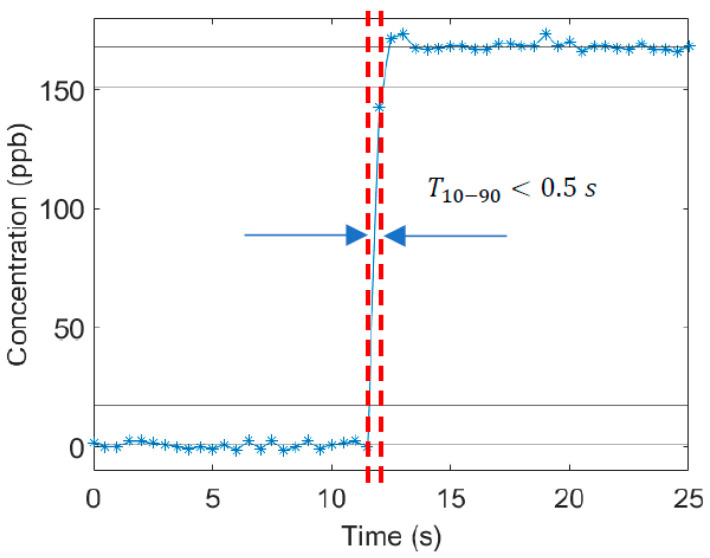
Sensor response to a sudden change in supplied ozone concentration [35].

Sensor time response is a key specification for atmospheric air quality measurements, in particular to faithfully capture geo-located signals from mobile sampling approaches (Section 1). Assuming adequate optical sensitivity, the time response of optical sensors is typically limited by the flow residence time (flush time) of the sample gas passing through the sampling cell. Our sensor design is based on a re-entrant optical cavity (as opposed to a multi-pass cavity where the beam should not overlap itself) meaning we can have a relatively small cell volume which gives lower residence times for a given pumping configuration (flowrate). For these measurements, we have employed a 40 cm long stainless-steel cell with a 1.05 cm inner diameter resulting in a total volume of ~0.035 L. The flowrate, with a compact pump, was 12.5 L per minute, yielding a (single-pass flush) residence time of ~0.3 s. Figure 6 shows the instrument’s temporal response to a step change in concentration under these conditions. The data yields a 10–90% settling time, *T*_10–90,_ of <~0.5 s (as shown in Figure 6) which compares favorably with the expectations and field requirements. 

### 3.5. Demonstrative Field Deployment

**Figure 7:**
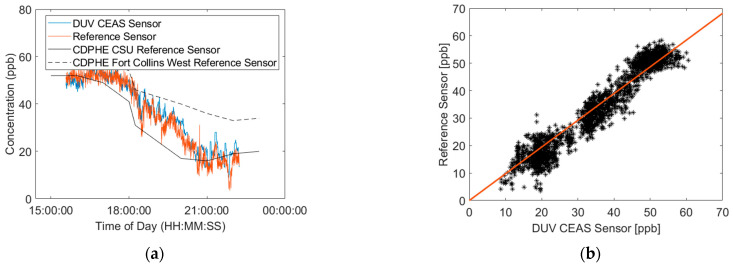
(**a**) Measurements of ozone from DUV-CEAS instrument and reference sensor along with two CDPHE monitors on 26 September 2022. (**b**) Scatter plot of DUV-CEAS sensor versus reference sensor.

Here, we present the results of an initial field deployment test with a co-located reference analyzer (2B Technologies Model 306, Broomfield, CO, USA) to show the ability of the DUV-CEAS sensor to sample air from real outdoor conditions. The pair of instruments was deployed in the second-floor laboratory of the Powerhouse Energy Campus (PEC) at Colorado State University (CSU) in Fort Collins, CO. Both instruments had similar inlets which were 4 mm diameter tubes, of a 4 m length, each protruding ~0.75 m out of a window roughly 6 m above the ground. The readings of both sensors were also compared against two Colorado Department of Public Health (CDPHE) monitors located at proximate locations in Fort Collins, CO (The Fort Collins West CDPHE sensor being ~5 km west of the PEC, and the CSU CDPHE sensor being ~1.5 km south of the PEC). Figure 7 shows the comparison between the sensors measuring ambient air in Fort Collins on 26 September 2022. The CSU sensor readings which were taken at a rate of 1 Hz were averaged to 10 s to match the output of the reference monitor for comparisons. A linear fit of the scatter plot yields a slope of 1.016 (R^2^ of 0.910), which is indicative of a good agreement between the sensors. These test results, sampling real urban air with time-varying O_3_, and showing agreement with the reference analyzer, support the basic spectroscopic approach and are promising for practical field use. Issues connected to spectral detection in the presence of other species are discussed in the conclusion. For outdoor mobile use, the instrument can be further ruggedized to deal with the effects of temperature variation and vibration, etc., as has been shown for other cavity-enhanced optical instruments, e.g., that in [10,42]. 

## 4. Conclusions

We have presented the design and testing of a cavity-enhanced absorption spectroscopic instrument, using the deep-ultraviolet output of a broadband laser-driven light source, for the measurement of ozone. The in situ (point) sensor has attractive specifications for ozone detection including an accuracy better than 2%, precision better than 1 ppb, and the possibility of fast detection at rates better than one measurement per second. The combination of high sensitivity and fast response derive from several aspects including the use of a bright DUV source to overlap with the strongest part of the absorption spectrum, the use of cavity enhancement (effective path length > 50 m), and finally the small cavity volume (which itself is enabled by the high spectral radiance of the LDLS). This combination of parameters exceeds what is available from many commercial analyzers, particularly semiconductor-based sensors, but also many optical analyzers, and should allow a range of ground-based field studies, including those conducted via mobile platforms, for source detection, identification, and quantification. Of course, the instrument can also be deployed at fixed locations including for applications such as industrial monitoring and process control (e.g., in connection with water and food sterilization).

Relative to the landscape of other ozone instruments, it is acknowledged that there are other instruments with even better parameters, which may be needed depending on the application details. It is interesting to specifically compare instruments based on DUV LEDs [28,29]. A notable difference is that LED-based instruments generally work over smaller spectral regions and use non-dispersive detectors, which invokes several tradeoffs. The non-dispersive detectors are less expensive; however, our spectrometer-based system may be better suited to warding off interference effects from other absorbing species (e.g., oxygen), since it analyzes a broader spectral region. The use of DUV-CEAS can also be more readily extended to the detection of other species, notably benzene, with absorption lines that are even further in the DUV range (e.g., ~235–265 nm for benzene) where LEDs tend to be lower-performing or less available [43]. In some measurement environments of interest, e.g., regions with emissions from oil and gas and/or urban regions with a myriad of emission sources, ambient air can have a complex composition including ozone and multiple VOCs [8,44]. Instrument performance in ambient air containing a matrix of VOC gases, with multiple absorbers potentially present in the spectral measurement region, is an area needing future research including the development of fitting approaches for the optimization of selectivity and detection limits. The CEAS sensor development presented, using a LDLS, can help pave the way forward for related instruments for the detection of benzene and the many other VOCs with DUV absorption. 

## Data Availability

Data are available upon request to the author.

## References

[1] Lu X., Zhang L., Shen L. (2019). Meteorology and Climate Influences on Tropospheric Ozone: A Review of Natural Sources, Chemistry, and Transport Patterns. Curr. Pollut. Rep..

[2] Nuvolone D., Petri D., Voller F. (2018). The effects of ozone on human health. Environ. Sci. Pollut. Res..

[3] Emberson L. (2020). Effects of ozone on agriculture, forests and grasslands. Philos. Trans. R. Soc. A Math. Phys. Eng. Sci..

[4] Colorado Department of Public Health and Environment, State of Colorado Technical Support Document for Recommended 8-Hour Ozone Designations, Air Pollution Control Division, Adopted 15 September 2016, Denver, Colorado. https://www.epa.gov/sites/default/files/2016-11/documents/co-rec-tsd.pdf.

[5] Thompson T.M., Shepherd D., Stacy A., Barna M.G., Schichtel B.A. (2017). Modeling to Evaluate Contribution of Oil and Gas Emissions to Air Pollution. J. Air Waste Manag. Assoc..

[6] Cheadle L.C., Oltmans S.J., Pétron G., Schnell R.C., Mattson E.J., Herndon S.C., Thompson A.M., Blake D.R., McClure-Begley A. (2017). Surface ozone in the Colorado northern Front Range and the influence of oil and gas development during FRAPPE/DISCOVER-AQ in summer 2014. Elem. Sci. Anthr..

[7] Gilman J.B. (2015). Oil and Gas VOC Emissions and Chemistry, E.C.S.L. Review, Editor. https://www.google.com.hk/url?sa=i&rct=j&q=&esrc=s&source=web&cd=&cad=rja&uact=8&ved=0CAIQw7AJahcKEwioxNifqIr_AhUAAAAAHQAAAAAQAg&url=https%3A%2F%2Fcsl.noaa.gov%2Freviews%2F2015%2Fpresentations%2F42Gilman.pdf&psig=AOvVaw3pn9ObVMsM5I3dLL9ku0Pi&ust=1684892252787906.

[8] Gilman J.B., Lerner B.M., Kuster W.C., de Gouw J.A. (2013). Source Signature of Volatile Organic Compounds from Oil and Natural Gas Operations in Northeastern Colorado. Environ. Sci. Technol..

[9] Albertson J.D., Harvey T., Foderaro G., Zhu P.P., Zhou X.C., Ferrari S., Amin M.S., Modrak M., Brantley H., Thoma E.D. (2016). A Mobile Sensing Approach for Regional Surveillance of Fugitive Methane Emissions in Oil and Gas Production. Environ. Sci. Technol..

[10] McHale L.E., Martinez B., Miller T.W., Yalin A.P. (2019). Open-path cavity ring-down methane sensor for mobile monitoring of natural gas emissions. Opt. Express.

[11] Caulton D.R., Li Q., Bou-Zeid E., Fitts J.P., Golston L.M., Pan D., Lu J., Lane H.M., Buchholz B., Guo X.H. (2018). Quantifying uncertainties from mobile-laboratory-derived emissions of well pads using inverse Gaussian methods. Atmos. Chem. Phys..

[12] Golston L.M., Aubut N.F., Frish M.B., Yang S.T., Talbot R.W., Gretencord C., McSpiritt J., Zondlo M.A. (2018). Natural Gas Fugitive Leak Detection Using an Unmanned Aerial Vehicle: Localization and Quantification of Emission Rate. Atmosphere.

[13] Sun K., Tao L., Miller D.J., Khan M.A., Zondlo M.A. (2014). On-Road Ammonia Emissions Characterized by Mobile, Open-Path Measurements. Environ. Sci. Technol..

[14] Arfire A., Marjovi A., Martinoli A. Mitigating Slow Dynamics of Low-Cost Chemical Sensors for Mobile Air Quality Monitoring Sensor Networks. Proceedings of the 2016 International Conference on Embedded Wireless Systems and Networks.

[15] Marjovi A., Arfire A., Martinoli A. High Resolution Air Pollution Maps in Urban Environments Using Mobile Sensor Networks. Proceedings of the 2015 International Conference on Distributed Computing in Sensor Systems (DCOSS).

[16] Lee J.K., Christen A., Ketler R., Nesic Z. (2017). A mobile sensor network to map carbon dioxide emissions in urban environments. Atmos. Meas. Tech..

[17] Hannun R.A., Swanson A.K., Bailey S.A., Hanisco T.F., Bui T.P., Bourgeois I., Peischl J., Ryerson T.B. (2020). A cavity-enhanced ultraviolet absorption instrument for high-precision, fast-time-response ozone measurements. Atmos. Meas. Tech..

[18] Sui N., Wei X., Cao S., Zhang P., Zhou T., Zhang T. (2022). Nanoscale Bimetallic AuPt-Functionalized Metal Oxide Chemiresistors: Ppb-Level and Selective Detection for Ozone and Acetone. ACS Sens..

[19] Snyder E.G., Watkins T.H., Solomon P.A., Thoma E.D., Williams R.W., Hagler G.S.W., Shelow D., Hindin D.A., Kilaru V.J., Preuss P.W. (2013). The Changing Paradigm of Air Pollution Monitoring. Environ. Sci. Technol..

[20] Castell N., Dauge F.R., Schneider P., Vogt M., Lerner U., Fishbain B., Broday D., Bartonova A. (2017). Can commercial low-cost sensor platforms contribute to air quality monitoring and exposure estimates?. Environ. Int..

[21] Wing R., Godin-Beekmann S., Steinbrecht W., McGee T.J., Sullivan J.T., Khaykin S., Sumnicht G., Twigg L. (2021). Evaluation of the new DWD ozone and temperature lidar during the Hohenpeißenberg Ozone Profiling Study (HOPS) and comparison of results with previous NDACC campaigns. Atmos. Meas. Tech..

[22] Axelsson H., Edner H., Galle B., Ragnarson P., Rudin M. (1990). Differential Optical Absorption Spectroscopy (DOAS) Measurements of Ozone in the 280–290 nm Wavelength Region. Appl. Spectrosc..

[23] Fiedler S.E., Hese A., Ruth A.A. (2003). Incoherent broad-band cavity-enhanced absorption spectroscopy. Chem. Phys. Lett..

[24] Berden G., Engeln R. (2009). Cavity Ring-Down Spectroscopy, Techniques and Applications.

[25] Gao R.S., Ballard J., Watts L.A., Thornberry T.D., Ciciora S.J., McLaughlin R.J., Fahey D.W. (2012). A compact, fast UV photometer for measurement of ozone from research aircraft. Atmos. Meas. Tech..

[26] Proffitt M.H., McLaughlin R.J. (1983). Fast-response dual-beam UV-absorption ozone photometer suitable for use on stratospheric balloons. Rev. Sci. Instrum..

[27] Washenfelder R.A., Wagner N.L., Dube W.P., Brown S.S. (2011). Measurement of Atmospheric Ozone by Cavity Ring-down Spectroscopy. Environ. Sci. Technol..

[28] Kalnajs L.E., Avallone L.M. (2010). A Novel Lightweight Low-Power Dual-Beam Ozone Photometer Utilizing Solid-State Optoelectronics. J. Atmos. Ocean. Technol..

[29] Gomez A.L., Rosen E.P. (2013). Fast response cavity enhanced ozone monitor. Atmos. Meas. Tech..

[30] Islam M., Ciaffoni L., Hancock G., Ritchie G.A.D. (2013). Demonstration of a novel laser-driven light source for broadband spectroscopy between 170 nm and 2.1 mu m. Analyst.

[31] Sachsenhauser M. Insights from Industry: Laser-Driven Light Sources and the Future of Photonics Innovation. https://www.azooptics.com/Article.aspx?ArticleID=2258.

[32] Energetiq. High Brightness, Broadband Light Source with Fiber-Coupled Output. https://www.energetiq.com/eq99xfc-fiber-coupled-broadband-light-source.

[33] Washenfelder R.A., Attwood A.R., Flores J.M., Zarzana K.J., Rudich Y., Brown S.S. (2016). Broadband cavity-enhanced absorption spectroscopy in the ultraviolet spectral region for measurements of nitrogen dioxide and formaldehyde. Atmos. Meas. Tech..

[34] Energetiq H. Laser-Driven Light Source LDLS. https://www.hamamatsu.com/content/dam/hamamatsu-photonics/sites/documents/99_SALES_LIBRARY/etd/LDLS_TLSZ1038E.pdf.

[35] Molina L.T., Molina M.J. (1986). Absolute absorption cross sections of ozone in the 185- to 350-nm wavelength range. J. Geophys. Res. Atmos..

[36] Thalman R., Zarzana K.J., Tolbert M.A., Volkamer R. (2014). Rayleigh scattering cross-section measurements of nitrogen, argon, oxygen and air. J. Quant. Spectrosc. Radiat. Transf..

[37] Rothman L.S., Gordon I.E., Babikov Y., Barbe A., Benner D.C., Bernath P.F., Birk M., Bizzocchi L., Boudon V., Brown L.R. (2013). The HITRAN2012 molecular spectroscopic database. J. Quant. Spectrosc. Radiat. Transf..

[38] Bogumil K., Orphal J., Homann T., Voigt S., Spietz P., Fleischmann O.C., Vogel A., Hartmann M., Kromminga H., Bovensmann H. (2003). Measurements of molecular absorption spectra with the SCIAMACHY pre-flight model: Instrument characterization and reference data for atmospheric remote-sensing in the 230–2380 nm region. J. Photochem. Photobiol. A Chem..

[39] Lee B.C., Huang W., Tao L., Yamamoto N., Gallimore A.D., Yalin A.P. (2014). A cavity ring-down spectroscopy sensor for real-time Hall thruster erosion measurements. Rev. Sci. Instrum..

[40] Huang H.F., Lehmann K.K. (2010). Long-term stability in continuous wave cavity ringdown spectroscopy experiments. Appl. Opt..

[41] Werle P., Mucke R., Slemr F. (1993). The Limits of Signal Averaging in Atmospheric Trace-Gas Monitoring by Tunable Diode-Laser Absorption-Spectroscopy (Tdlas). Appl. Phys. B Photophysics Laser Chem..

[42] Lack D.A., Richardson M.S., Law D., Langridge J.M., Cappa C.D., McLaughlin R.J., Murphy D.M. (2012). Aircraft Instrument for Comprehensive Characterization of Aerosol Optical Properties, Part 2: Black and Brown Carbon Absorption and Absorption Enhancement Measured with Photo Acoustic Spectroscopy. Aerosol Sci. Technol..

[43] Thorlabs. LEDs on Metal-Core PCBs Deep UV LEDs (265–340 nm). https://www.thorlabs.com/newgrouppage9.cfm?objectgroup_id=6071.

[44] de Gouw J.A., Gilman J.B., Kim S.W., Alvarez S.L., Dusanter S., Graus M., Griffith S.M., Isaacman-VanWertz G., Kuster W.C., Lefer B.L. (2018). Chemistry of Volatile Organic Compounds in the Los Angeles Basin: Formation of Oxygenated Compounds and Determination of Emission Ratios. J. Geophys. Res. Atmos..

